# Correlation between cystathionine β-synthase T883C genetic polymorphism and primary hypertension

**DOI:** 10.3892/etm.2014.1799

**Published:** 2014-06-23

**Authors:** YING ZHANG, HONG WANG, HUAN-WEN SUN, YU-LAN CHEN, JU-YAN OUYANG, YU WANG, LING WANG, XIANG-YANG ZHANG

**Affiliations:** Department of Cadre Ward, First Affiliated Hospital of Xinjiang Medical University, Urumqi, Xinjiang 830011, P.R. China

**Keywords:** primary hypertension, gene, homocysteine, cystathionine β-synthase, Kazakh

## Abstract

The present study aimed to investigate the correlation between cystathionine β-synthase (CBS) T833C polymorphisms and primary hypertension. A case-control study was conducted by genotyping the representative variation in 545 hypertensive individuals (aged 49.23±7.56 years) and 500 normotensive individuals (aged 49.90±10.01 years). The T833C genetic polymorphisms of the CBS enzyme were detected in all subjects by amplification refractory mutation system polymerase chain reaction (PCR) analysis. The CBS T833C polymorphism was successfully genotyped in the general population with a sample size of 1,045 (545+500) individuals. The genotypic and allelic frequency distributions of the CBS T833C polymorphism were not significantly different between the hypertensive and normotensive groups (P>0.05). The CC genotype was significantly different (P<0.05) from the CT and TT genotypes in terms of body mass index (BMI), and the levels of triglycerides (TG) and homocysteine (Hcy). Multiple logistic regression analysis revealed that BMI, total cholesterol (TC) level, smoking, plasma Hcy level and a family history of hypertension were the independent risk factors for hypertension in the population studied. The results indicate that the level of plasma Hcy was a risk factor for hypertension in the population studied. However, the mutation of the CBS T833C gene was not concluded to be an important hereditary factor for influencing the level of plasma Hcy.

## Introduction

A number of environmental and clinical risk factors are associated with essential hypertension (EH), including dietary intake of sodium, alcohol intake, lack of exercise, a poor diet, obesity, insulin-resistant diabetes and hyperlipidemia (1). Although these factors explain a substantial proportion of hypertension susceptibility, it is estimated that up to 60% of the variation in hypertension risk is due to the genetic makeup of an individual (2).

The association between hypertension and hyperhomocysteinemia and the risk of cardiovascular disease (CVD) has been previously studied (3). The three major enzymes involved in homocysteine (Hcy) metabolism are methylenetetrahydrofolate reductase (MTHFR), methionine synthase (MS) and cystathionine β-synthase (CBS). A study has revealed that the 677T MTHFR allele is associated with increased levels of plasma Hcy in ethnic Europeans (4). A study of a population in the Midwestern region of the USA demonstrated that a 68-bp insertion in the CBS gene (844ins68) and the A2756G transition of the MS gene were associated with low levels of plasma Hcy (5). To the best of our knowledge, a total of 132 mutations in CBS have been identified to date with the majority of these being missense mutations. The most frequent among these is I278T (c.833T>C), commonly found in Caucasians, for which the clinical manifestation is mild and vitamin B6-responsive (6). While there has been major focus on the association between CBS T833C polymorphism and cerebral arterial thrombosis in the Chinese population (7,8), few studies have investigated its association with hypertension.

The Kazakhs, a nomad population that inhabit, among other areas, the north of Xinjiang province in northwest China, are characterized by a higher prevalence of hypertension and higher levels of blood pressure (BP) compared with other ethnic populations residing in the same area (9). Since hypertension is a common disease among the Kazakh people, the present study concentrated on the CBS T833C polymorphism in order to investigate whether it was correlated with hypertension in Kazakh patients.

## Materials and methods

### Subjects

The sample size for the Kazakh hypertensive group [systolic blood pressure (SBP) ≥140 mmHg or diastolic blood pressure (DBP) ≥90 mmHg)] was 545 (male:female ratio, 409:136; mean age, 49.23±7.56 years; SBP, 161.18±23.62 mmHg; DBP, 96.46±10.99 mmHg). The sample size for the Kazakh normotensive control group (SBP <140 mmHg and DBP <90 mmHg) was 540 (male:female ratio, 385:155; mean age, 49.90±10.01 years, SBP, 120.92±11.85 mmHg; DBP, 76.91±7.94 mmHg). The subjects were selected from a population-based cross sectional study performed during January March 2010 and August 2012 among the Kazakh population. The study concentrated on isolated populations in a homogeneous environment. The criteria for the selection of hypertensive subjects were a SBP ≥140 mmHg, DBP ≥90 mmHg or anti-hypertension treatment (10). The criteria for the selection of normotensive control subjects was a SBP <140 mmHg, DBP <90 mmHg and no history of anti-hypertensive medication. Individuals with secondary hypertension (estimated by their history, examinations and laboratory evaluation), serious arrhythmia, coronary heart disease, liver and renal insufficiency, heart failure, cerebrovascular accidents, acute and chronic infectious diseases, cancer, and those using contraceptives were excluded from the current study. All Kazakh subjects who participated in the present study were residents of the Xinjiang autonomous region of China, which has an established Kazakh population who are considered to be less affected by the migration of the Han Chinese. The characteristics of the subjects analyzed in the present study are summarized in Table I. Written consent was obtained from all subjects prior to data collection and measurements. The current study was approved by the Ethics Committee of the First Affiliation of Xinjiang Medical University (Xinjang, China).

### Study design and procedures

The blood pressure measurements were performed by trained and certified observers three times for each subject following at least 10 min of rest in a sitting position. Weight and height were measured using standard techniques with the participants in light clothing and barefoot. Body mass index (BMI) was calculated as weight (kg)/height (m^2^). Blood samples from the patients were collected in the morning following overnight fasting from the antecubital vein. The samples were divided into aliquots, separated within 30 min and stored at −80°C until transportation for analysis. An enzyme-linked immunosorbent assay (ELISA) was used to measure the levels of Hcy (11). In addition to performing a routine blood examination that included lipid profiling, and glucose level, blood/urine electrolyte and anthropometric measurements, all subjects completed a set of questionnaires that included questions on demographic information, personal history, family history of disease and lifestyle.

### DNA isolation

Genomic DNA was extracted from the blood sample of each subject using the PAXgene^™^ gene blood DNA kit (Qiagen, Hilden, Germany/BD Biosciences, Franklin Lakes, NJ, USA).

### Detection of the CBS T833C polymorphism

**Figure 1:**
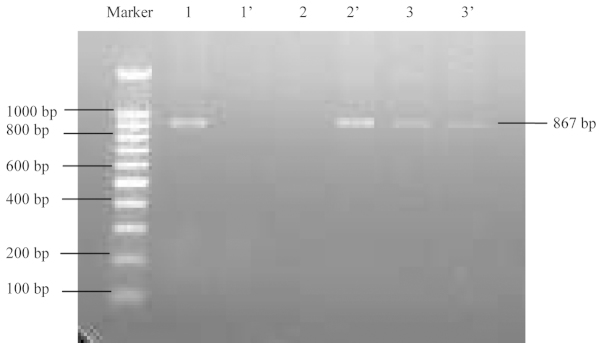
CBS T833C gene polymorphisms. Lanes 1 and 1′, 2 and 2′, 3 and 3′ were the same DNA samples; 1–3 were the P1/P2 polymerase chain reaction (PCR) products; 1′-3′ were the P1/P3 PCR products. 1, TT genotype; 2, CT genotype; 3, CC genotype. CBS, cystathionine β-synthase.

The CBS T833C polymorphism was analyzed through amplification refractory mutation system polymerase chain reaction (PCR) analysis (12). The forward primer (P1) was (5′-GGA GAA GTG TCC TGG ATG CA-3′), the wild type reverse primer (P2) was (5′-CCC TTC GGG ATC CAC CCC AA-3′) and the mutant type reverse primer (P3) was (5′-CCC TT CGG GAT CC AC CCC AG-3′). The PCR reaction was conducted with a final volume of 20 μl in two tubes. The 10 μl reaction mix (Tarcine BioMed, Inc., Beijing, China), 0.5 μl each primer, 8 μl ddH_2_O and 1 μl genomic DNA were used for the amplification. Cycling conditions included an initial denaturation at 94°C for 5 min followed by 35 cycles with a fast denaturation at 94°C for 60 sec, an annealing step at 60°C for 45 sec and an extension step at 72°C for 60 sec, with a final incubation for 7 min at 72°C. The amplification reaction was followed by digestion with the reaction enzyme, MspA1I (Fermentas Inc., Beijing, China) at 37°C for 24 h and electrophoresis on a 2% agarose gel. The PCR product was 867 base pairs in size (Fig. 1). CBS TT and CC genotypes were identified as only one band (P1 vs. P2 display respectively), while the CT genotype was identified as two bands (P1 and P2 displays).

### Statistical analysis

Statistical analysis was performed using SPSS software, version 16.0 (SPSS, Inc., Chicago, IL, USA). The Student’s t-test or one-way analysis of variance (ANOVA) was used to compare numeric variables. The χ^2^ test was used to determine any deviation in genotype distribution from Hardy-Weinberg equilibrium and any differences in the frequency of the CBS T833C polymorphism. The correlation between risk factors and hypertension was assessed using logistic regression analysis. The odds ratio (OR) with a 95% confidence interval (CI) was determined. P<0.05 was considered to indicate a statistically significant difference.

## Results

### Profile of the clinical characteristics of the study groups

The demographic and clinical characteristics of the study population are shown in Table I. Age and genders were comparable in the two groups. The levels of smoking and drinking, BMI, SBP, DBP, fasting plasma glucose (FPG), total cholesterol (TC), triglycerides (TG), low density lipoprotein (LDL) and Hcy were significantly higher in the hypertensive group compared with those in the normotensive group (P<0.05). The levels of uric acid (UA) and high density lipoprotein (HDL) were not observed to be statistically different between the two groups (P>0.05).

### CBS T883C genotype and allele frequency distribution in the Kazakh population

The distribution of the CBS T883C genotype identified in the two groups did not significantly deviate from the Hardy-Weinberg equilibrium. CBS T883C genotypes in the hypertensive and control subjects are shown in Table II. No statistical difference in genotype distribution was identified between the hypertensive and control groups (P=0.36) and no significant difference in allele frequency of the CBS T883C polymorphism was observed between the two groups (68.9 vs. 65.7%, respectively, P=0.12).

### Comparing the clinical characteristics in the CBS T883C genotype

The clinical characteristics in the CBS T883C genotypes of the Kazakh subjects are shown in Table III. BMI, TG and Hcy were significantly different for the CBS CC genotype than for the CBS TT and CT genotypes (P<0.05). SBP, DBP, FPG, UA, TC, HDL and LDL were not significantly different between the three genotypes.

### Multivariate analysis of the CBS T833C polymorphism in the Kazakh population

Table IV shows the multivariate analysis of risk factors relating to hypertension and CBS T833C under a conditional logistic regression model. The present study revealed that BMI, TC, smoking, level of plasma Hcy and a family history of hypertension were independently correlated with a significant predisposition to hypertension following adjustments for related risk factors including age, gender and drinking. The CBS T833C polymorphism was not found to be correlated with a significant predisposition to hypertension.

## Discussion

Hcy is an amino acid derived from the demethylation of methionine. Hyperhomocysteinemia is associated with an increased risk of several complex diseases, including CVD (13). Hao *et al* (14) revealed a high prevalence of hyperhomocysteinemia in Chinese adults, with 18% having Hcy concentrations >16.0 mmol/l. This was especially prevalent among males in the north of China where 28% of adults (40% of males) had Hcy levels above this, a four-fold increase compared with that in the south. Published data suggest that concentrations of plasma Hcy vary depending on age and have significant ethnic and gender differences (15–17). Since the fruit and vegetable intake of people in China relies on local and seasonal produce, the intake of green-leaf vegetables with a high folate content is much higher in the south than in the north of the country. The Kazakhs, as a nomad population, inhabit the north of Xinjiang province in northwest China and 99% of them are herdsmen. Their diet consists of a higher intake of salt than of green-leaf vegetables. The present study identified that not only traditional risk factors, including the levels of smoking and drinking, BMI, SBP, DBP, FPG, UA, TC, TG, HDL and LDL, but also Hcy were significantly higher in subjects with hypertension than in those with normotension (17.19±6.11 vs. 13.77±5.66 μmol/l, respectively). Thus, close monitoring to control cardiovascular risk factors is recommended in the Kazakh population.

CBS, one of the three major enzymes involved in Hcy metabolism, catalyzes the initial step of the transsulfuration pathway, which condenses Hcy and serine to produce cystathionine and ultimately cysteine (18). Kozich and Kraus (19) used a bacterial expression system and western blot analysis to demonstrate that unstable CBS subunits were formed by clones containing the I278T (c.833T>C) mutation. They suggested that the substitution of hydrophobic isoleucine by a more hydrophilic threonine residue may affect the CBS conformation or the interaction of the subunits, which may result in an unstable tetrameric CBS. The human CBS gene is located at chromosome 21q22.3 (20). A total of 132 mutations in CBS have been identified to date, with the majority of these being missense mutations. Furthermore, 92 disease-associated mutations have been identified in the CBS gene (21). The most frequently occurring of these is I278T (c.833T>C), which substitutes threonine for isoleucine at codon 278. A number of studies investigating the CBS T833C polymorphism have focused on CVD (22,23). A meta-analysis by Ding *et al* indicated that the CBS T833C polymorphism is associated with an increased risk of stroke, and that the C allele is likely to be an important risk factor for stroke (24). Fewer studies, however, have investigated its association with hypertension, especially in the Kazakh population.

To the best of our knowledge, the current study is the first study to demonstrate a correlation between the CBS T833C allele and hypertension in the Kazakh population (21). The present study confirmed the presence of the CBS T833C polymorphism in the Kazakh population and detected the frequency of the TT, CT and CC genotypes, which were 51.9, 33.9 and 14.1%, respectively, in the hypertensive group, and 48.1, 35.2 and 15.4%, respectively, in the normotensive group. No statistically significant difference was identified in the genotype distribution between the two groups. Furthermore, the frequency of the T allele genotype in subjects with hypertension (68.9%) was not statistically different from that of subjects with normotension (65.7%), which demonstrated that a single gene mutation does not have a significant influence on hypertension in the Kazakh population. When comparing the clinical characteristics among different CBS T833C genotypes, the present study demonstrated significant correlations not only between CBS T833C and BMI and TG, but also between CBS T833C and Hcy. The C allele was correlated with increased BMI, TG and Hcy. Multivariate logistic analyses revealed that BMI, TC, smoking, level of plasma Hcy and a family history of hypertension, but not CBS T833C, were independent risk factors for hypertension in the Kazakh population in Xinjiang. This indicated that not only the T833C mutation of the CBS gene but other factors, including age, smoking and the levels of TG and LDL, were associated with Hcy metabolism.

Numerous studies have focused on investigating the association between the methylene tetrahydrofolate reductase (MTHFR) C677T genetic polymorphism and primary hypertension in the Chinese population. Zong *et al* (25) demonstrated that the homozygous C677T mutation of the MTHFR gene was a major heredity factor for increased levels of Hcy and H-type hypertension in the Chinese population, and that this was also related to gender. However, other studies did not observe the same association between the Hcy gene polymorphisms and primary hypertension (26–28). Though the study by Zong *et al* revealed that Hcy was an independent risk factor of primary hypertension in the Kazakh population, it did not reveal that the CBS T833C mutations were associated with primary hypertension in the Kazakh population, which further demonstrates that a single factor and/or gene locus is responsible for hypertension, in particular, in the Kazakh population in Xinjiang. In addition, due to the complexity of the Hcy metabolic pathway, the Hcy metabolism may have been affected by certain interactions between the three enzymes involved. Therefore, further large-scale studies are required to be carried out among the Kazakh population in the future, in particular examining polygenes and multiple mutations.

## Figures and Tables

**Table I:** Comparison of clinical characteristics in the Kazakh subjects.

Characteristic	Hypertension	Normotension	t/χ^2^	P-value
N (male/female)	545 (409/136)	540 (385/155)	1.93	0.16
Age (years)	49.23±7.56	49.90±10.01	0.56	0.57
Smoking (n/%)	390/71.6	325/60.2	15.61	0.02
Drinking (n/%)	387/71.0	330/61.1	11.85	0.01
BMI (kg/m^2^)	27.31±3.94	24.98±3.28	4.84	0.01
SBP (mmHg)	161.18±23.62	120.92±11.85	15.72	0.01
DBP (mmHg)	96.46±10.99	76.91±7.94	15.25	0.01
FPG (mmol/l)	5.83±1.17	5.56±1.67	2.06	0.04
UA (mmol/l)	294.69±59.20	282.27±34.87	1.88	0.06
TC (mmol/l)	4.48±0.98	3.72±1.25	5.35	0.01
TG (mmol/l)	3.38±1.54	2.69±1.53	3.50	0.01
HDL (mmol/l)	1.21±0.33	1.19±0.24	0.04	0.97
LDL, (mmol/l)	2.73±0.65	2.10±0.58	7.85	0.01
Hcy (μmol/l)	17.19±6.11	13.77±5.66	4.24	0.01

BMI, body mass index; SBP, systolic blood pressure; DBP, diastolic blood pressure; FPG, fasting blood-glucose; UA, uric acid; TC, total cholesterol; TG, triglyceride; HDL, high density lipoprotein; LDL, low density lipoprotein; Hcy, homocysteine.

**Table II:** Genotype distribution and allele frequency of the CBS T833C polymorphism in the Kazakh population.

Genotype/allele	Hypertension (n=545)	Control (n=540)	χ^2^-value	P-value
TT	283 (51.9%)	260 (48.1%)	2.03	0.36
CT	185 (33.9%)	190 (35.2%)		
CC	77 (14.1%)	90 (15.4%)		
T	751 (68.9%)	710 (65.7%)	2.46	0.12
C	339 (31.1%)	370 (34.3%)		

CBS, cystathionine β-synthase; TT, homozygotic TT genotype; CT, heterozygotic CC genotype; CC, homozygotic CC genotype.

**Table III:** Comparison of clinical characteristics in the CBS T833C genotypes in the Kazakh subjects.

Characteristic	TT	TC	CC	F-value	P-value
BMI (kg/m^2^)	25.58±3.39	26.73±4.12	28.19±4.07^a^	6.85	0.01
SBP (mmHg)	145.57±27.78	144.62±28.60	142.06±27.25	0.21	0.82
DBP (mmHg)	88.43±13.35	89.14±14.40	86.88±13.83	0.33	0.72
FPG (mmol/l)	5.65±0.88	5.80±1.21	5.79±0.76	0.66	0.51
UA (mmol/l)	288.87±39.05	295.29±67.09	276.97±35.65	1.59	0.21
TC (mmol/l)	4.02±1.20	4.32±1.16	4.29±0.95	1.97	0.14
TG (mmol/l)	2.43±1.43	3.08±1.28	3.34±1.28^a^	9.19	0.01
HDL (mmol/l)	1.18±0.31	1.19±0.32	1.04±0.27	1.69	0.19
LDL (mmol/l)	2.43±0.65	2.56±0.73	2.44±0.75	0.93	0.39
Hcy (μmol/l)	15.36±5.73	15.62±6.32	16.31±6.70^a^	5.95	0.01

BMI, body mass index; SBP, systolic blood pressure; DBP, diastolic blood pressure; FPG, fasting blood-glucose; UA, uric acid; TC, total cholesterol; TG, triglyceride; HDL, high density lipoprotein; LDL, low density lipoprotein; Hcy, homocysteine; TT, homozygotic TT genotype; CT, heterozygotic CC genotype; CC, homozygotic CC genotype.

a P<0.05 vs. CBS TT and TC genotypes.

**Table IV:** Multivariate analysis for the CBS T833C polymorphism in the Kazakh population according to a conditional logistic regression model.

Variable	β-coefficient	SE	Wald	95% CI	P-value
BMI	0.26	0.07	12.75	1.29 (1.12, 1.49)	0.01
Age	0.05	0.03	2.90	1.05 (0.99, 1.10)	0.09
Gender	0.24	0.50	0.23	1.26 (0.48, 3.36)	0.63
Family history of hypertension	2.10	0.58	13.07	8.19 (2.62, 25.65)	0.01
Smoking	2.98	0.81	13.70	9.68 (4.06, 25.39)	0.01
Drinking	0.63	0.70	0.81	1.88 (0.48, 7.45)	0.37
FPG	−0.13	0.28	0.21	0.88 (0.51, 1.51)	0.65
TC	0.55	0.22	6.39	1.72 (1.13, 2.63)	0.01
TG	−0.22	0.65	0.73	1.06 (0.48, 2.36)	0.54
Hcy	0.81	0.37	4.74	2.25 (1.09, 4.69)	0.03
CBS T833C	0.04	0.03	1.58	1.04 (0.98, 1.11)	0.21

CI, confidence interval; BMI, body mass index; FPG, fasting blood-glucose; TC, total cholesterol, TG: triglyceride; Hcy, homocysteine; CBS, cystathionine β-synthase. SE, standard error.

## References

[1] Fowdar JY, Lason MV, Szvetko AL, Lea RA, Griffiths LR (2012). Investigation of homocysteine-pathway-related variants in essential hypertension. Int J Hypertens.

[2] Singh M, Mensah GA, Bakris G (2010). Pathogenesis and clinical physiology of hypertension. Cardiol Clin.

[3] Towfighi A, Markovic D, Ovbiagele B (2010). Pronounced association of elevated serum homocysteine with stroke in subgroups of individuals: a nationwide study. J Neurol Sci.

[4] Chambers JC, Obeid OA, Refsum H, Ueland P, Hackett D, Hooper J, Turner RM, Thompson SG, Kooner JS (2000). Plasma homocysteine concentrations and risk of coronary heart disease in UK Indian Asian and European men. Lancet.

[5] Tsai MY, Bignell M, Yang F, Welge BG, Graham KJ, Hanson NQ (2000). Polygenic influence on plasma homocysteine: association of two prevalent mutations, the 844ins68 of cystathionine β-synthase and A2756G of methionine synthase, with lowered plasma homocysteine levels. Atherosclerosis.

[6] Kruger WD, Wang L, JHee KH, Singh RH, Elsas LJ (2003). Cystathionine β-synthase deficiency in Georgia (USA): correlation of clinical and biochemical phenotype with genotype. Hum Mutat.

[7] Wu JM, Wang TG, Li YQ, Song XW, Liu YY, Yun HR, Zhong ZY, Zhou TH (2004). Genetic mutations of homocysteine metabolism related enzymes in patients with ischemic stroke. Yi Chuan.

[8] Lv YL, Li JZ, Mi MR, Wei HX, Liu XD (2010). Correlation of cystathionine β synthase gene polymorphisms and risk of cerebral arterial thrombosis in Chinese population: a meta-analysis. Chin J Evid-based Med.

[9] Li NF, Zhang JH, Chang JH, Yang J, Wang HM, Zhou L, Luo WL (2011). Association of genetic variations of the prostasin gene with essential hypertension in the Xinjiang Kazakh population. Chin Med J (Engl).

[10] Liu LS, Writing Group of 2010 Chinese Guidelines for the Management of Hypertension (2011). 2010 Chinese guidelines for the management of hypertension. Zhonghua Xin Xue Guan Bing Za Zhi.

[11] Frantzen F, Faaren AL, Alfheim I, Nordhei AK (1998). Enzyme conversion immunoassay for determining total homocysteine in plasma or serum. Clin Chem.

[12] Pepe G, Vanegas OC, Rickards O, Giusti B, Comeglio P, Brunelli T, Marcucci R, Prisco D, Gensini GF, Abbate R (1999). World distribution of the T833C/844INS68 CBS in cis double mutation: a reliable anthropological marker. Hum Genet.

[13] Malinowska A, Chmurzynska A (2009). Polymorphism of genes encoding homocysteine metabolism-related enzymes and risk for cardiovascular disease. Nutr Res.

[14] Hao L, Ma J, Zhu J, Stampfer MJ, Tian Y, Willett WC, Li Z (2007). High prevalence of hyperhomocysteinemia in Chinese adults is associated with low folate, vitamin B-12, and vitamin B-6 status. J Nutr.

[15] Must A, Jacques PF, Rogers G, Rosenberg IH, Selhub J (2003). Serum total homocysteine concentrations in children and adolescents: results from the third National Health and Nutrition Examination Survey (NHANES III). J Nutr.

[16] Jacques PF, Rosenberg IH, Rogers G, Selhub J, Bowman BA, Gunter EW, Wright JD, Johnson CL (1999). Serum total homocysteine concentrations in adolescent and adult Americans: results from the third National Health and Nutrition Examination Survey. Am J Clin Nutr.

[17] Malinow MR, Ducimetiere P, Luc G, Evans AE, Arveiler D, Cambien F, Upson BM (1996). Plasma homocyst(e)ine levels and graded risk for myocardial infarction: findings in two populations at contrasting risk for coronary heart disease. Atherosclerosis.

[18] Miles EW, Kraus JP (2004). Cystathionine β-synthase: structure, function, regulation, and location of homocystinuria-causing mutations. J Biol Chem.

[19] Kozich V, Kraus JP (1992). Screening for mutations by expressing patient cDNA segments in *E. coli*: homocystinuria due to cystathionine β-synthase deficiency. Hum Mutat.

[20] Münke M, Kraus JP, Ohura T, Francke U (1988). The gene of cystathionine β-synthase (CBS) maps to the subtelomeric region on human chromosome 21q and to proximal mouse chromosome 17. Am J Hum Genet.

[21] Kraus JP, Janosík M, Kozich V, Mandell R (1999). Cystathionine β-synthase mutations in homocystinuria. Hum Mutat.

[22] Carnicer R, Navarro MA, Arbonés-Mainar JM, Arnal C (2007). Genetically based hypertension generated through interaction of mild hypoalphalipoproteinemia and mild hyperhomocysteinemia. J Hypertens.

[23] Grobelny BT, Ducruet AF, DeRosa PA, Kotchetkov IS, Zacharia BE (2011). Gain-of-function polymorphisms of cystathionine β-synthase and delayed cerebral ischemia following aneurysmal subarachnoid hemorrhage. J Neurosurg.

[24] Ding R, Lin S, Chen D (2012). The association of cystathionine β synthase (CBS) T833C polymorphism and the risk of stroke: a meta-analysis. J Neurol Sci.

[25] Zong YH, Li XY, Chen GL, Xu XP, Li JP, Huo Y, Wei JH, Zhao LS (2011). Homocysteine level and N5, 10-methylenetetrahydrofolate reductase gene polymorphism in hypertensive population. J Clin Cardiol.

[26] Wang H, Wu GZ, Zhang Y, Zhang XY, Chen YZ, Sikaer A (2010). Association of homocysteine and its metabolic enzyme genes polymorphisms with essential hypertension in Xinjiang Kazakhs. J Clin Rehabil Tissue Eng Res.

[27] Wang H, Zhang Y, Zhang XY, Wu GZ, Chen YL, Sikaer A (2010). Relationship of homocysteine and methionine synthase A2756G polymorphisms with essential hypertension in Kazak nationality in Xinjiang. J Chin Pract Diagn Ther.

[28] Zhang Y, Wang H, Zhang XY, Wang L, Wu GZ (2012). Relationship between homocysteine, methylene tetrahydrofolate reductase C677T polymorphisms and essential hypertension in Kazak nationality in Xinjiang. J Clin Cardiol.

